# Prevalence and determinants of hypertension among urban slum dwellers in Bangladesh

**DOI:** 10.1186/s12889-022-14456-3

**Published:** 2022-11-11

**Authors:** Sabuj Kanti Mistry, Md. Belal Hossain, Mahmood Parvez, Rajat Das Gupta, Amit Arora

**Affiliations:** 1 BRAC James P Grant School of Public Health, BRAC University, 28, 6th Floor, Medona Tower, Bir Uttam AK Khandakar Rd, Dhaka, 1213 Bangladesh; 2 Centre for Primary Health Care and Equity, University of New South Wales, Kensington, NSW 2052 Australia; 3 ARCED Foundation, 13/1 Pallabi, Mirpur-12, Dhaka, 1216 Bangladesh; 4 Department of Public Health, Daffodil International University, Dhaka, 1207 Bangladesh; 5 School of Population and Public Health, University of British Columbia, 2206 East Mall, Vancouver, BC V6T 1Z3 Canada; 6 Department of Epidemiology and Biostatistics, Arnold School of Public Health, University of South Carolina, Columbia, SC 29208 USA; 7 School of Health Sciences, Western Sydney University, Campbelltown Campus, Locked Bag 1797, Penrith, NSW 2751 Australia; 8 Translational Health Research Institute, Western Sydney University, Locked Bag 1797, Penrith, NSW 2751 Australia; 9 Discipline of Child and Adolescent Health, Sydney Medical School, Faculty of Medicine and Health, The University of Sydney, Westmead, NSW 2145 Australia; 10 Oral Health Services, Sydney Local Health District and Sydney Dental Hospital, NSW Health, Surry Hills, NSW 2010 Australia; 11 Health Equity Laboratory, Campbelltown, NSW 2560 Australia

**Keywords:** Hypertension, Urban slum, Risk factors, Bangladesh, Blood pressure

## Abstract

**Background:**

In low- and middle- income countries such as Bangladesh, urban slum dwellers are particualry vulnerable to hypertension due to inadequate facilities for screening and management, as well as inadequate health literacy among them. However, there is scarcity of evidence on hypertension among the urban slum dwellers in Bangladesh. The present study aimed to determine the prevalence and factors associated with hypertension among urban slum dwellers in Bangladesh.

**Methods:**

Data were collected as part of a large-scale cross-sectional survey conducted by Building Resources Across Communities (BRAC) between October 2015 and January 2016. The present analysis was performed among 1155 urban slum dwellers aged 35 years or above. A structured questionnaire was adminstered to collect data electronically and blood pressure measurements were taken using standardised procedures. Binary logistic regression with generalized estimating equation modelling was performed to estimate the factors associated with hypertension.

**Results:**

The prevalence of hypertension was 28.3% among urban slum dwellers aged 35 years and above. In adjusted analysis, urban slum dwellers aged 45–54 years (AOR: 1.64, 95% CI: 1.17–2.28), 55–64 years (AOR: 2.47, 95% CI: 1.73–3.53) and ≥ 65 years (AOR: 2.34, 95% CI: 1.47–3.72), from wealthier households (AOR: 1.94, 95% CI: 1.18–3.20), sleeping < 7 h per day (AOR: 1.87, 95% CI: 1.39–2.51), who were overweight (AOR: 1.53, 95% CI: 1.09–2.14) or obese (AOR: 2.34, 95% CI: 1.71–3.20), and having self-reported diabetes (AOR: 3.08, 95% CI: 1.88–5.04) had an increased risk of hypertension. Moreover, 51.0% of the participants were taking anti-hypertensive medications and 26.4% of them had their hypertension in control.

**Conclusions:**

The findings highlight a high burden of hypertension and poor management of it among the slum dwellers in Bangladesh requiring a novel approach to improve care. It is integral to effectively implement the available national non-communicable disease (NCD) control guidelines and redesign the current urban primary health care system to have better coordination.

## Background

Hypertension is a major risk factor of cardiovascular diseases (CVDs) [1]. Globally, aound 1.13 billion people are diagnosed with hypertension and majority of them are residing in low- and middle- income countries (LMICs) [2]. Recent studies reported that the prevalence of hypertension ranged from 18.0% to 47.3% among the adult population in LMICs [3–6]. Like other LMICs, the prevalence of hypertension is high in Bangladesh. According to the most recent data [7], the prevalence of hypertension was 25.2% and 19.8% respectively in urban and rural areas of Bangladesh among the adults aged 18–69 years.

Previous studies conducted in LMICs including Bangladesh [4, 5, 8–11] identified several risk factors associated with hypertension. Some of these risk factors included higher age, overweight and/or obesity, sedentary lifestyle, and pre-existing co-morbidities.

UN-Habitat defined slum as “a heavily populated urban area characterised by substandard housing and squalor” [12]. Globally, around one billion people live in slum settlements [13], of which around 130 million lives in South Asian countries [13]. Like many other South Asian countries, most of the urban growth has been taken place in urban slums of Bangladesh [14]. It has been estimated that around 29 million people, which is around 55% of the total urban population in Bangladesh is currently residing in urban slums [15].

Most of the urban slum dwellers in Bangladesh exhibit poor living conditions and are deprived of basic health care services, making them vulnerable to a range of illnesses including maternal and child health related problems as well as chronic conditions such as hypertension [15–19]. Poverty contributes to lack of access to basic health care services among these population with concomitant increases in the risk of hypertension and uncontrolled blood pressure. This might be due to lack of blood pressure screening, poor management of hypertension, and absence of health education [7]. Previous studies carried out in the slum settlements of LMICs [20–23] reported a high prevalence of hypertension among slum dwellers. For example, Banerjee et al. (2016) found that the prevalence of hypertension was 42% among the adult population in slums of Kolkata, India [20]. Likewise, the prevalence of hypertension was 21% among urban slum dwellers in Brazil [22].

However, there is scarcity of evidence on the prevalence and risk factors for hypertension among the adult population from slum areas of Bangladesh. Therefore, the current study aimed to investigate the prevalence and associated factors of hypertension among the adult slum dwellers (aged 35 years and above) in Bangladesh.

## Subjects and methods

### Study setting and participants

**Fig. 1:**
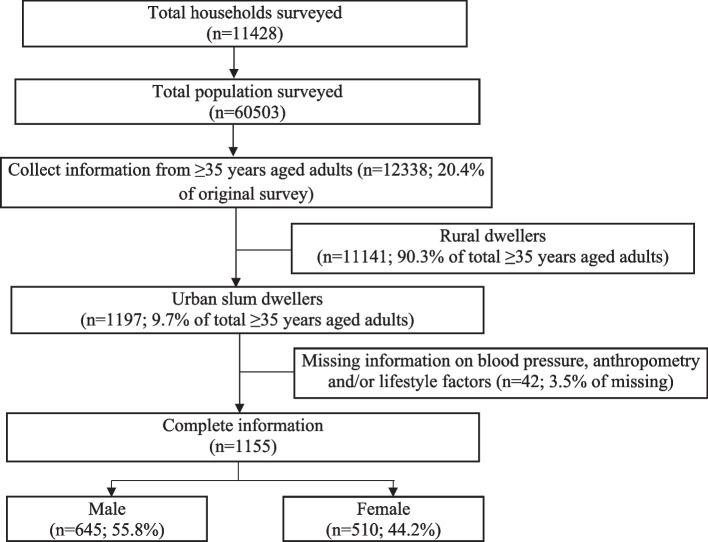
Flowchart showing the selection of adults aged 35 years of more for the present study

Data used in this study were collected as part of a large-scale cross-sectional survey conducted between October 2015 and January 2016 in Bangladesh. The main survey was conducted by Building Resources Across Communities (BRAC) in rural areas and urban slums of Bangladesh. Briefly, 180 enumerations areas (EAs) from rural areas, and 30 EAs from urban slums were randomly selected to provide statistically reliable estimates of the key health indicators for the rural areas and urban slums separately, where an EA is typically a ward in slum areas, which is the lowest administrative unit of urban areas in Bangladesh. A complete list of unions (rural) and wards (urban slum) from the most recent Population and Housing Census of Bangladesh were collected and used as a sampling frame for the first stage of sampling. In the second stage of sampling, the EAs per administrative zone were selected following the probability proportional to size technique with a systematic random sample of five households. Starting from the north-west corner of an EA with a systematic random sample of five households, we selected on an average of 54 households per EA [24]. In the present study, we considered 1197 adults aged 35 years and above residing in the slum households. In these slums BRAC operates its health and nutrition intervention where BRAC’s frontline community health workers deliver behavioral change communication messages, particularly on maternal and child health care, essential health services and nutrition among the slum dwellers through door-to-door visits [25]. Inclusion criteria for the present study were age being 35 years or above and residing in slum settlements for more than six months. A total of 42 participants were also excluded due to missing and/or inconsistent information; 1155 completed the questionnaire accurately and included in the final analysis (Fig. 1).

### Data collection tools and techniques

Household level socio-demographic information were collected through face-to-face interviews with each respondent using a structured questionnaire. The questionnaire was pre-tested in Gazipur, a suburb of Dhaka, and the feedback was incorporated into the final version of the questionnaire. A total of 110 skilled interviewers, having prior experience of conducting large-scale healthcare surveys were recruited and trained for data collection. Data collection was carried out electronically in ODK (Open Data Kit), an Android-based open-source mobile platform software [26]. The ODK can be used to collect data both online and offline and can be used by the people with limited educational qualifications. Its unique features such as GPS tracker and short message service (SMS) enable the real-time data collection monitoring [27]. A multi-layered monitoring system comprising of field supervisors as the first layer, followed by the researchers, and finally by statisticians positioned at the head office, was employed to validate, standardize and maintain data quality.

### Outcome measurement

The WHO guidelines were followed to measure the blood pressure of the study participants [28]. The blood pressure was measured by trained field interviewers with a standardized calibrated mercury sphygmomanometer on the right arm in a seated posture with feet on the floor and arm supported at the heart level. Two blood pressure measurements were taken using the ALPK2 Blood Pressure Monitor- initial measurement was taken after 5 min of rest and second measurement was performed after 2 min resting interval of the first measurement, and the average of the two measurements was recorded. The individuals were classified according to the Seventh Report of the Joint National Committee on Prevention, Detection, Evaluation, and Treatment of High Blood Pressure (JNC 7): who had a systolic blood pressure of (SBP) ≥ 140 mm Hg or a diastolic blood pressure (DBP) ≥ 90 mm Hg or were taking any prescribed medication to control blood pressure were categorized as hypertensive [29].

### Explanatory variables

Socio-demographic variables considered in this study were: age of the respondents (35–44, 45–54, 55–64, and ≥ 65 years), gender (male/female), level of education (no education, primary incomplete, primary/secondary incomplete, and secondary or higher), occupation (unemployed/employed), marital status (currently married/single), locality (city corporation/municipality), and household wealth status (poorest, poorer, middle, richer, and richest). Lifestyle and clinical factors were: current smoker or smokeless tobacco user (no/yes), daily sleep duration (< 7 h, 7–9 h, and ≥ 10 h), physical activity (inactive, less active, and moderate to highly active), body mass index (underweight, normal weight, overweight, and obese), and self-reported diabetes status (no/yes).

Locality of the urban slums were classified as either city corporation or municipality which are the distinct local government systems of urban areas in Bangladesh. The city corporation locality covers the slums in the 12 city corporation areas (basically in the largest cities in the country), while slums located in municipality areas are those situated in urban areas other than city corporations. Household wealth index was constructed using factor analysis [30, 31] of household-level key socioeconomic variables: types of wall, floor and roof of the house; ownership of a radio, television, computer, bicycle, mobile phone/telephone, refrigerator, wardrobe, table, chair, watch/clock, bed, sewing machine, bicycle, motor vehicle, livestock and access to solar power and electricity. The wealth index was categorized into poorest, poorer, middle, richer, and richest based on cut-point values of the wealth score (≤ 20%, 21–40%, 41–60%, 61–80% and > 80%).

The participants were considered as ‘current smoker or current smokeless tobacco’ user if they consumed tobacco or smokeless tobacco in the past 30 days [28]. Sleeping time was categorized based on the recommendation of the National Sleep Foundation [32]. Physical activity was measured through a modified activity questionnaire that comprises routine daily tasks such as commuting, occupational tasks or household activities, and purposeful health-enhancing movements/activities. Participants who did not do any physical activity were classified as inactive; those who spent 75 min of vigorous-intensity physical activity, or 150 min of moderate-intensity physical activity, or an equivalent combination of vigorous- and moderate-intensity activity in a week were categorized as high activity; others were classified as less active. Body mass index (BMI) was calculated as weight in kg/(height in meter)^2^, which was categorized using Asian cut-off: underweight (BMI < 18.5), normal (BMI: 18.5–22.9), overweight (BMI: 23.0–24.9), and obese (BMI≥ 25.0) [33]. The height was measured with the participants standing on a board having a wooden base and a movable headpiece to the nearest 0·1 cm. On the otherhand, the weight was measured to the nearest 0·1 kg with an electronic bathroom scale [34]. The individuals with known type 2 diabetes were defined as those who had been diagnosed earlier by a registered physician. Trained interviewers verified any self-reported diabetes case thorough checking any available laboratory reports and/or prescriptions.

### Statistical analysis

Descriptive statistics were performed to show the distribution of variables. The chi-square test was used to compare the prevalence of hypertension among different socio-demographic and lifestyle factors. Binary logistic regression model was applied to assess the relationship between hypertension and socio-demographic and lifestyle factors. Notably, data were hierarchical, and there was cluster (EA) to cluster variation in terms of the hypertension prevalence. In addition, adults from the same cluster could share certain types of unobserved cultural and environmental factors. Hence, we adjusted the clustering effect in logistic regression. In this case, we run the binary logistic regression with a generalized estimating equation by assuming an exchangeable correlation structure among clusters. We considered an independent, unstructured, autoregressive order 1 and exchangeable correlation structure among clusters. The model with the smallest quasi-likelihood was considered the best model. In our data, we observed approximately similar quasi-likelihood values for all models, and thus, we reported the results with an exchangeable correlation structure. The curde odds ratio (COR), adjusted odds ratio (AOR), and 95% confidence interval (CI) were reported. The variables with *P* < 0.25 were only included in the multivariable model [35]. For the final model, 5% was considered as the statistical significance level. We also performed the model diagnostics, such as calculating the area under the curve, checking the multicollinearity using variance inflation factor (VIF), an Omnibus test to see if the final model as a whole is statistically significant than the intercept-only model, overall model performance using Nagelkerke R^2^, and goodness of fit of the final model using Hosmer–Lemeshow test. All analyses were performed using the statistical software package STATA (Version 15.0) (StataCorp, College Station, TX, USA).

## Results

### Sociodemographic characteristics

Table 1 shows the sociodemographic characteristics of the study population. Among 1,155 participants, 42.4% were aged < 45 years, 55.8% were male, 53.9% had no formal education, 62.9% were from city corporation area, and 40.8% were from poorer and poorest households.

**Table 1:** Bivariate analysis of sociodemographic and lifestyle characteristics with hypertension among adult urban slum dwellers in Bangladesh (*N* = 1,155)

Characteristics	Total (*N* = 1,155)	% No hypertension (*N* = 828)	% Hypertension (*N* = 327)	*P*
Overall	100.00	71.69	28.31	*-*
Age in years				
35–44	490 (42.42)	81.84	18.16	< 0.001
45–54	298 (25.80)	69.46	30.54	
55–64	240 (20.78)	61.67	38.33	
≥ 65	127 (11.00)	56.69	43.31	
Gender				
Male	645 (55.84)	76.12	23.88	< 0.001
Female	510 (44.16)	66.08	33.92	
Level of education				
No formal education	622 (53.85)	71.22	28.78	0.925
Primary incomplete	172 (14.89)	73.84	26.16	
Primary/secondary incomplete	264 (22.86)	71.59	28.41	
Secondary or higher	97 (8.40)	71.13	28.87	
Occupation				
Unemployed	534 (46.23)	64.79	35.21	< 0.001
Employed	621 (53.77)	77.62	22.38	
Current marital status				
Married	974 (84.33)	74.02	25.98	
Single	181 (15.67)	59.12	40.88	
Household wealth status ^a^				
Poorest	169 (20.00)	73.16	26.84	0.014
Poorer	177 (20.78)	73.75	26.25	
Middle	176 (19.39)	78.57	21.43	
Richer	155 (20.61)	65.13	34.87	
Richest	151 (19.22)	68.02	31.98	
Locality				
City corporation	726 (62.86)	73.19	26.81	
Municipality	429 (37.14)	70.80	29.20	0.383
Current smoker or smokeless tobacco user ^b^				
No	416 (36.02)	69.95	30.05	0.326
Yes	739 (63.98)	72.67	27.33	
Daily sleep hours				
Less than 7 h	294 (25.45)	64.29	35.71	< 0.001
7–9 h	772 (66.84)	75.39	24.61	
More than 9 h	89 (7.71)	64.04	35.96	
Physical activity ^c^				
Inactivity	329 (28.48)	72.64	27.36	0.643
Less activity	602 (52.12)	72.09	27.91	
Moderate to high activity	224 (19.39)	69.20	30.80	
BMI ^d^				
Underweight	100 (8.66)	79.00	21.00	< 0.001
Normal	529 (45.80)	78.26	21.74	
Overweight	233 (20.17)	70.39	29.61	
Obese	293 (25.37)	58.36	41.64	
Self-reported diabetes				
No	1072 (92.81)	74.16	25.84	< 0.001
Yes	83 (7.19)	39.76	60.24	

^a^ Household wealth index was constructed using factor analysis of household-level key socioeconomic variables and categorized into poorest, poorer, middle, richer, and richest based on cut-point values of the wealth score (≤ 20%, 21–40%, 41–60%, 61–80% and > 80%)

^b^ The participants were considered as ‘current smoker or current smokeless tobacco’ user if they consumed tobacco or smokeless tobacco in the past 30 days

^c^ Physical activity was measured through a modified activity questionnaire that comprises routine daily tasks such as commuting, occupational tasks or household activities, and purposeful health-enhancing movements/activities. Participants who did not do any physical activity were classified as inactive; those who spent 75 min of vigorous-intensity physical activity, or 150 min of moderate-intensity physical activity, or an equivalent combination of vigorous- and moderate-intensity activity in a week were categorized as high activity; others were classified as less active

^d^ Body mass index (BMI) was calculated as weight in kg/(height in meter)^2^ and categorized using Asian cut-off of underweight (BMI < 18.5), normal (18.5–22.9), overweight (23.0–24.9), and obese (≥ 25.0)

### Prevalence of hypertension

**Fig. 2:**
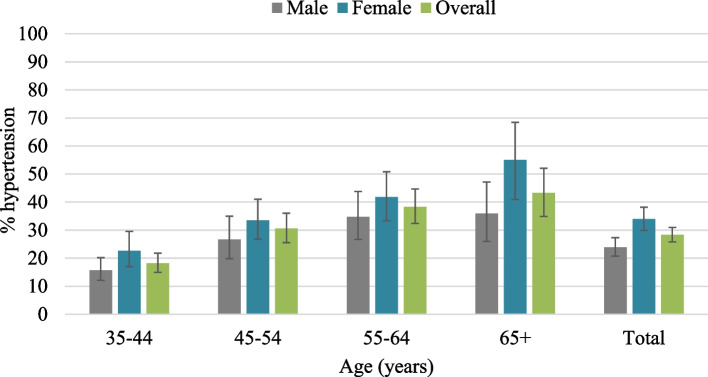
Prevalence of hypertension by age and gender

**Fig. 3:**
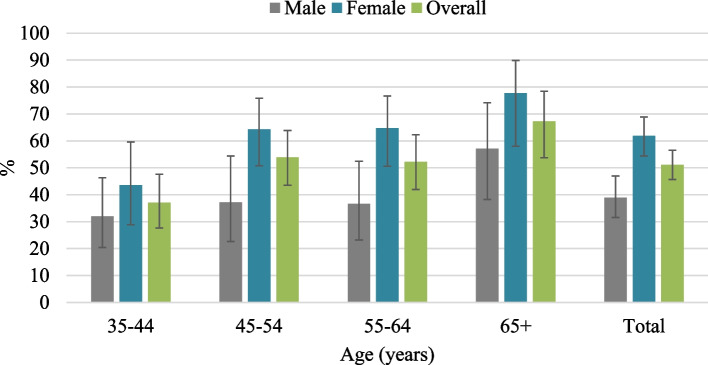
Among participants with hypertension, the distribution of those taking anti-hypertensive medications by age and gender (*n* = 327)

**Fig. 4:**
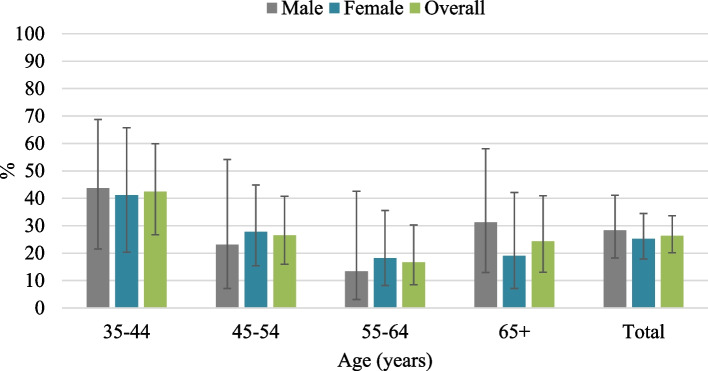
Hypertension control among the participants with hypertension who were taking anti-hypertensive medications by age and gender (*n* = 167)

The overall prevalence of hypertension was 28.3% (Fig. 2). The prevalence was significantly higher in females than males (33.9% versus 23.9%) and among participants aged 65 years and above compared to those who were aged 35–44 years (43.3% versus 18.2%). The prevalence of hypertension was also significantly higher among those who were unemployed compared to those who were employed (35.2% versus 22.4%). Likewise, the prevalence was significantly higher among the participants who were currently single than those who were married (40.9% versus 26.0%). Also, the prevalence of hypertension was significantly higher among those who were from the wealthier households compared to those who were from the poorest households (poorest 26.8%; richer 34.9%; richest 31.9%). It was also found that the prevalence was significantly higher among the participants who slept less than 7 h per day (35.7%) or more than 9 h per day (36.0%), compared to to those who slept 7–9 h per day (24.6%). Moreover, the prevalence was significantly higher among those who were obese compared to the participants who had normal weight (41.6% versus 21.7%), and among those who had self-reported diabetes compared to those who did not have self-reported diabetes (60.2% versus 25.8%) (Table 1). Among the participants with hypertension, 51.0% were taking anti-hypertensive medications. This percentage was significantly higher among females than males but was consistent across the age groups (Fig. 3). Moreover, around one-fourth of the participants with hypertension who were taking anti-hypertensive medications (26.4%) had their hypertension in control i.e., having SBP of < 140 mm Hg or DBP < 90 mm Hg. Hypertension control was significantly higher among relatively younger participants (35–44 years), however, no significant difference was observed in terms of gender (Fig. 4).

### Factors associated with hypertension

Table 2 shows the factors associated with hypertension among urban slum dwellers in Bangladesh. In the unadjusted analysis, age, gender, occupation, current marital status, household wealth status, sleep, BMI, and diabetes were significantly associated with hypertension (*P* < 0.05). Respondent's age, household wealth status, sleep, BMI, and self-reported diabetes remained significant in the adjusted analysis.

**Table 2:** Factors associated with hypertension among adult urban slum dwellers in Bangladesh

Characteristics	Unadjusted		Adjusted	
	COR (95% CI)	*P*	AOR (95% CI)	*P*
Age in years				
35–44	Ref		Ref	
45–54	1.81 (1.32–2.49)	< 0.001	1.64 (1.17–2.28)	0.004
55–64	2.87 (2.07–3.99)	< 0.001	2.47 (1.73–3.53)	< 0.001
≥ 65	3.28 (2.18–4.93)	< 0.001	2.34 (1.47–3.72)	< 0.001
Gender				
Male	Ref		Ref	
Female	1.59 (1.24–2.03)	< 0.001	1.04 (0.71–1.51)	0.856
Level of education				
No formal education	Ref		NA	
Primary incomplete	0.85 (0.58–1.22)	0.376		
Primary/secondary incomplete	0.97 (0.71–1.32)	0.834		
Secondary or higher	0.96 (0.60–1.53)	0.872		
Occupation				
Unemployed	Ref		Ref	
Employed	0.52 (0.41–0.67)	< 0.001	0.73 (0.50–1.06)	0.103
Current marital status				
Married	Ref		Ref	
Single	1.89 (1.37–2.61)	< 0.001	1.18 (0.81–1.72)	0.375
Household wealth status ^a^				
Poorest	Ref		Ref	
Poorer	1.30 (0.81–2.08)	0.273	1.29 (0.79–2.11)	0.310
Middle	1.15 (0.70–1.89)	0.582	1.16 (0.69–1.97)	0.573
Richer	2.10 (1.32–3.35)	0.002	1.94 (1.18–3.20)	0.009
Richest	1.62 (1.00–2.63)	0.052	1.36 (0.80–2.29)	0.251
Locality				
Municipality	Ref		NA	
City corporation	1.14 (0.73–1.77)	0.576		
Current smoker or smokeless tobacco user ^b^				
No	Ref		Ref	
Yes	0.84 (0.65–1.09)	0.200	0.94 (0.71–1.25)	0.673
Daily sleep hours				
Less than 7 h	1.73 (1.31–2.28)	< 0.001	1.87 (1.39–2.51)	< 0.001
7–9 h	Ref			
More than 9 h	1.35 (0.83–2.22)	0.228	1.15 (0.68–1.94)	0.593
Physical activity ^c^				
Inactivity	Ref		NA	
Less activity	1.10 (0.82–1.47)	0.536		
Moderate to high activity	1.14 (0.78–1.67)	0.485		
BMI ^d^				
Underweight	0.94 (0.57–1.54)	0.801	1.02 (0.62–1.67)	0.950
Normal	Ref		Ref	
Overweight	1.45 (1.04–2.03)	0.029	1.53 (1.09–2.14)	0.014
Obese	2.38 (1.76–3.22)	< 0.001	2.34 (1.71–3.20)	< 0.001
Self-reported diabetes				
No	Ref		Ref	
Yes	4.18 (2.64–6.64)	< 0.001	3.08 (1.88–5.04)	< 0.001

*COR:* Crude odds ratio, *AOR:* Adjusted odds ratio, *CI:* Confidence interval, *BMI:* Body mass index. NA – not considered that variable in the adjusted model

^a^ Household wealth index was constructed using factor analysis of household-level key socioeconomic variables and categorized into poorest, poorer, middle, richer, and richest based on cut-point values of the wealth score (≤ 20%, 21–40%, 41–60%, 61–80% and > 80%)

^b^ The participants were considered as ‘current smoker or current smokeless tobacco’ user if they consumed tobacco or smokeless tobacco in the past 30 days

^c^ Physical activity was measured through a modified activity questionnaire that comprises routine daily tasks such as commuting, occupational tasks or household activities, and purposeful health-enhancing movements/activities. Participants who did not do any physical activity were classified as inactive; those who spent 75 min of vigorous-intensity physical activity, or 150 min of moderate-intensity physical activity, or an equivalent combination of vigorous- and moderate-intensity activity in a week were categorized as high activity; others were classified as less active

^d^ Body mass index (BMI) was calculated as weight in kg/(height in meter)^2^ and categorized using Asian cut-off of underweight (BMI < 18.5), normal (18.5–22.9), overweight (23.0–24.9), and obese (≥ 25.0)

**Fig. 5:**
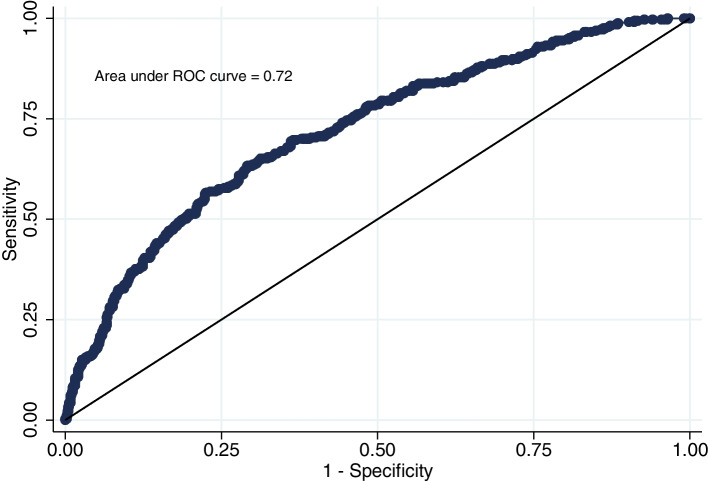
Area under the receiver operating characteristic (ROC) curve for exploring the factors associated with hypertension among adult urban slum dwellers in Bangladesh

In the adjusted analysis, the odds of hypertension was significnalty higher among the participants aged 45–54 years (AOR: 1.64, 95% CI: 1.17–2.28), 55–64 years (AOR: 2.47, 95% CI: 1.73–3.53), and ≥ 65 years (AOR: 2.34, 95% CI: 1.47–3.72) compared to those aged 35–44 years. Participants from the richer households were more likely to be hypertensive than those from the poorest households (AOR: 1.94, 95% CI: 1.18–3.20). Moreover, sleeping less than 7 h per day was associated with higher odds of hypertension compared to sleeping 7–9 h per day (AOR: 1.87, 95% CI: 1.39–2.51). The odds of hypertension was 1.5 times higher among those who were overweight (AOR: 1.53, 95% CI: 1.09–2.14), and 2.3 times higher among those who were obese (AOR: 2.34, 95% CI: 1.71–3.20). Furthermore, participants with self-reported diabetes were more than three times odds of being hypertensive than participants without self-reported diabetes (AOR: 3.08, 95% CI: 1.88–5.04). In the final model, the area under the curve value was 0.72 (Fig. 5), meaning good discrimination; all VIF values were < 3 meaning no multicollinearity; the log-likelihood Chi-square of 145.9 with *P* < 0.001 of the Omnibus test indicates the significance of the final model over the intercept-only model; Nagelkerke R^2^ value of 0.11; and statistically insignificant Hosmer–Lemeshow test (*P* = 0.277) indicates good fit of the final model.

## Discussion

The present study investigated the prevalence and risk factors of hypertension among adults aged 35 years and over in urban slum areas of Bangladesh. More than one-quarter of the participants in this study were hypertensive. There is limited evidence on the prevalence of hypertension among urban slum dwellers in Bangldesh [36, 37]. However, according to the most recent STEPwise approach to surveillance (STEPS) survey conducted in Bangladesh (2018) data [38], the prevalence of hypertension was 25.2% among the urban slum dwellers which is close to what is reported among the slum dwellers in this study. A recent study conducted in rural Bangladesh also reported a similar (25.9%) prevalence of hypertension among the adult population [39]. The prevalence of hypertension was higher than those reported in slums from Brazil (21%) [22] and Kenya (12.3%) [23], but lower than those from Nigeria (38.2%) [21] and Kolkata (42%) [20].

The present study also identified several modifiable risk factors such as household wealth status, diabetes status, sleep duration, and BMI as signicant determinants of hypertension among the urban slum dwellers which are important to consider while designing an intervention to address the problem. The odds of hypertension was higher among those from wealthier households, which was also observed in studies conducted in slum areas of other LMICs [20, 21]. This positive association between wealth status and hypertension in LMICs can be explained by the theory that rural–urban migration, economic prosperity, and urbanization increases the prevalence of modifiable risk factors for hypertension such as overweight and obesity, sedentary lifestyle, and excessive caloric, fat, alcohol and salt intake [40]. Nevertheless, it is important to note that the wealth index variable in our study was created using the principal component analysis (PCA) of household assets. In practice, asset-based wealth and income can differ, so the asset-based wealth index may not meaningfully provide estimates of absolute income measures. However, asset-based wealth index can reflect the socio-economic status of the household, which is validated in Bangladesh and widely used, particularly in demographic and health survey [41]

Daily sleep duration was identified as a significant predictor of hypertension in the current study. The risk of hypertension was higher among those who reported sleeping < 7 h hours daily. Previous studies have shown that short sleep duration was found to be independently associated with weight gain and hypertension [42, 43]. Based on the evidence generated by the systematic reviews, the National Sleep Foundation recommends a sleep duration of 7–9 h/day for adults aged 18–64 years [44]. Short sleep duration may lead to hypertension through several biological pathways including increased adiposity, imbalance in hormones, dysfunction in metabolism and disruption in the circadian rhythmicity [45]. When the sleep is disrupted, it might lead to autonomic imbalance and thus increase in the sympathetic activity and decrease in the parasympathetic activity during sleep [45, 46]. This autonomic dysfunction leads to development of hypertension [45, 47]. Also, short sleep duration is associated with hormonal imbalance including low Leptin secretion, and high Ghrelin secretion, which leads to overweight and obesity which in turn causes hypertension [48].

In this study we observed that only half of the hypertensive patients were taking antihypertsive medications and this was higher among females. A recent study carried out in Bangladesh also reported similar findings [49]. The present study also found that, only one-quarter of the study participants who were on antihypertensive medications had their hypertension in control. This is relatively lower than that was documented in the latest Bangladesh Demographic and Health Survey 2017–18 [50], which reported that 33.8% of the population had their blood pressure controlled. However, previous studies conducted in slums of Kolkata [20] and Kenya [23] reported a lower percentage of people (12.0% and 21.5% respectively) with antihypertensive medications having their blood pressure controlled. This poor BP control among the slum dwellers could be for several reasons including lack of early detection, poor adherence to medications, lack of follow-ups after the initiation of medicaions [20, 51].

The health promotion programs in Bangladesh aimed to prevent hypertension, should focus on high-risk populations including urban slum dwellers. While there are available guidelines for the control and management of non-communicable diseases (NCDs) in Bangladesh [52, 53], they were not adequately implemented [54, 55]. Also, urban primary health care system is disjointed as it is currently under the Ministry of Local Government, Rural Development and Co-operatives (MoLGRD&Co) instead of Ministry of Health and Family Welfare (MOFHW) and there is a lack of coordination between them [56]. We recommend establishing a well-coordinated urban primary health care system with provisions for flexible hours in the outpatient department. The ‘NCD corner’ in the rural primary health care facilities can also be replicated in the urban primary health care system [57]. Moreover, the lessons learnt from other successful health programs in the urban slums (i.e., MANOSHI project of BRAC) can be translated in addressing the burden of hypertension and other NCDs using implementation science approach [55, 58].

The present study has several strengths to be highlighted. We used a robust sampling protocol to evaluate the risk factors of hypertension among urban slum dwellers in Bangladesh. However, this study has certain limitations. Firstly, the findings of the present study cannot be generalized for all slum areas of Bangladesh as the study was carried out in urban slums where BRAC operates its health and nutrition interventions. Second, being cross-sectional in nature, the study could not establish causality due to lack of temporal evidence between hypertension and its determinants. Thirdly, dietary salt intake was not assessed, and energy intake were not measured, which are established risk factors for hypertension. Fourth, it would have been interesting to directly compare the findings with those of non-slum population, but we were not able to do so due to limited data. Fifth, there was possibility of subjective reporting bias on data like exercise and sleep duration. Moreover, we used data which was collected during 2015–16 and thus a bit older. However, this is the latest available data collected on the urban slum dwellers in Bangladesh. The other surveys did not segregate data on urban slums.

## Conclusion

The present study highlighted that the prevalence of hypertension was high among the adult urban slum dwellers in Bangladesh. It was also revealed that the prevalence was high among those who were aged, obese, suffering from diabetes and who slept more than 7 h a day. Moreover, the study also found that the slum dwellers had poor hypertension management. Overall, hypertension appears as a high burden for slum dwellers with poor control and management, which requires a novel approach to improve care. Proper implementation of the national NCD control guidelines and establishment of a well-coordinated urban primary health care system would be of value in this regard.

## Data Availability

The dataset is owned by BRAC and has restriction to share it publicly. However, the datasets is available from the corresponding author on reasonable request and after getting permission from BRAC.
